# Assessing Alzheimer’s Disease and Related Dementia risk in American Samoa using the Talanoa research framework

**DOI:** 10.1002/alz.091031

**Published:** 2025-01-09

**Authors:** Vaatausili Tofaeono

**Affiliations:** ^1^ American Samoa Community Cancer Coalition, Pago Pago American Samoa

## Abstract

**Background:**

The American Samoa population has high prevalence for Alzheimer’s Disease and Related Dementia (ADRD) risk factors (e.g. tobacco use, obesity, hypertension, and diabetes), however ADRD risk has not been defined or assessed. The *Puipui Malu Manutu* (PMM) Study (RF1AG075904) is accomplishing this by assessing knowledge, health literacy, readiness, and resiliency and vulnerability factors using culturally adapted instruments in a sample of 1098 adults aged 50 and above. Additionally, Indigenous populations have unique worldview perspectives and epistemologies that have not been widely accepted in mainstream academia. Therefore, we decolonized Westernized research approaches by incorporating the *Talanoa* framework into the discussion and decision making of the ADRD risk profile each participant. The *Talanoa* framework is rooted in Samoan cultural norms to eliminate power, building trusting relationships, and is conducted face‐to‐face in individual or group settings. *Talanoa* is culturally grounded in four unique perspectives: alofa (love between the participant and researcher), fa’aaloalo (respect), tautau (service to the community), and malamalama (knowledge gained for the community).

**Method:**

We established a **Consensus Group** comprised of the PMM Multiple Principal Investigators, a Samoan Nurse Practitioner, a Samoan clinical Psychologies, and a Samoan Medical Doctor to define the ADRD risk The *Talanoa* framework guided the Consensus Group, with the PMM study staff and ADRD risk definition, to openly discuss and decide ADRD risk in participants in a culturally safe manner.

**Result:**

First, a definition of ADRD risk using cut offs and ranges of subjective and objective data gathered by various cognitive assessment tools was created. Second, preliminary results of 26 participants have shown 61.5% are at high risk. Diet and lack of physical activity are contributing risk factors. Further results will be made available at the time of presentation for the AAIC.

**Conclusion:**

Determining ADRD risk will assist public health programs to prioritize risk factors in education and awareness efforts. Decolonizing concepts and paradigms of ADRD research is a first step to ensuring Indigenous values are recognized to achieve a culturally safe environment and build trust for research in our community.

